# Preoperative [18F]FDG-PET Metabolic Patterns and Surgical Outcome in Mesial Temporal Lobe Epilepsy: A Retrospective SPM-Assisted Atlas-Based ROI Analysis

**DOI:** 10.3390/diagnostics16152327

**Published:** 2026-07-24

**Authors:** Gökhan Pek, İrem Yıldırım, Ümit Özgür Akdemir, Lütfiye Özlem Atay, Ali Yusuf Öner, Erhan Bilir

**Affiliations:** 1 Department of Neurology, Gazi University Faculty of Medicine, Ankara 06500, Türkiye; 2 Department of Nuclear Medicine, Gazi University Faculty of Medicine, Ankara 06500, Türkiye; 3 Department of Radiology, Gazi University Faculty of Medicine, Ankara 06500, Türkiye

**Keywords:** mesial temporal lobe epilepsy, hippocampal sclerosis, FDG-PET, epilepsy surgery, Statistical Parametric Mapping

## Abstract

**Background:** Interictal [18F]FDG-PET is commonly used for temporal lateralization in mesial temporal lobe epilepsy (mTLE). Whether atlas-based quantitative PET measures differ according to surgical outcome among otherwise concordant unilateral MRI-positive mTLE patients remains uncertain. This study compared preoperative FDG-PET ROI measures between 2-year Engel outcome groups using SPM-assisted spatial normalization followed by AAL atlas-based ROI analysis. **Methods:** Among 319 screened temporal lobe epilepsy surgery patients, 94 had evaluable preoperative FDG-PET images, and 62 patients with at least 2 years of postoperative follow-up were included in the total cohort. The primary quantitative analysis was restricted to 54 unilateral MRI-positive patients with hippocampal atrophy and mesial temporal sclerosis on MRI (Engel I, *n* = 45; Engel II–IV, *n* = 9). Cerebellum-normalized AAL ROI uptake ratios were compared using Mann–Whitney U tests in conventional left/right and ipsilateral/contralateral analyses. FDR-adjusted q values, effect sizes, and Hodges–Lehmann median differences with 95% confidence intervals were calculated. Reference-region and global mean normalization sensitivity analyses were also performed. **Results:** In the unilateral MRI-positive subgroup, 10 of 80 conventional left/right ROIs showed unadjusted differences between Engel I and Engel II–IV patients, predominantly involving left frontal-orbitofrontal, medial frontal, middle cingulate, and inferior parietal regions. After ipsilateral/contralateral transformation, eight ROIs showed unadjusted group differences, mainly in contralateral frontal-orbitofrontal regions, with additional cingulate and inferior parietal involvement. Uptake ratios were lower in Engel II–IV patients in all candidate ROIs. However, none of the ROI findings survived FDR correction. Raw cerebellar uptake did not differ between outcome groups, and global mean normalization did not yield FDR-corrected ROI findings. **Conclusions:** Quantitative ROI analysis identified several extratemporal metabolic differences between postoperative outcome groups; however, none remained significant after correction for multiple comparisons. These findings should therefore be considered exploratory and hypothesis-generating rather than evidence that quantitative FDG-PET can discriminate surgical outcomes. Larger, adequately powered cohorts are needed before the potential clinical utility of quantitative FDG-PET analysis in presurgical evaluation can be determined.

## 1. Introduction

According to the International League Against Epilepsy (ILAE), drug-resistant epilepsy is defined as “failure of adequate trials of two tolerated and appropriately chosen and used AED schedules (whether as monotherapies or in combination) to achieve sustained seizure freedom” [1]. In cases where seizure control cannot be achieved with drug treatment, surgery is considered; after surgery, 66–70% of patients are seizure-free at short-term (<5 years) follow-up [2]. However, a relevant subgroup of patients continues to have seizures despite apparently concordant presurgical findings. Chassoux et al. reported that although class I outcome was achieved in most patients with mTLE and hippocampal sclerosis, only a subgroup remained completely seizure-free in the long term, and surgical failure was related to metabolic patterns that extended beyond the focal anteromesial temporal region [3]. Similarly, Cahill et al. showed that metabolic patterns differed according to the side of mTLE and that contralateral temporal hypometabolism was associated with an unfavorable outcome in right mTLE [4]. These observations suggest that patients with apparently similar temporal lobe epilepsy may still differ in the extent and organization of their epileptic network.

FDG-PET is an established component of presurgical evaluation in drug-resistant focal epilepsy. Its conventional clinical role is to support localization and lateralization, especially when MRI is negative, equivocal, or discordant with electroclinical data. In large multicenter cohorts, FDG-PET has been shown to be particularly useful in temporal lobe epilepsy, where concordant PET hypometabolism contributes to surgical decision-making [5]. However, in routine clinical practice, FDG-PET is often interpreted primarily as a lateralizing test. Reports typically emphasize whether hypometabolism is ipsilateral and temporal in distribution. This approach is clinically practical, but it may underestimate the prognostic relevance of extratemporal or contralateral metabolic abnormalities.

More recent work has further framed mTLE as a network disorder. Cho et al. used graph theoretical analysis of FDG-PET and found significant differences in global and regional metabolic networks between seizure-free and non-seizure-free patients after temporal lobectomy [6]. Strýček et al. similarly showed that metabolic connectivity differed according to the lateralization of hippocampal sclerosis and surgical outcome; unfavorable outcome was related to decreased metabolic connectivity involving both hippocampi, the ipsilesional frontal operculum, and the contralesional insula, whereas favorable outcome involved decreased metabolic connectivity in the orbitofrontal and ipsilesional temporal networks [7]. These findings indicate that metabolic abnormalities outside the temporal lobe may reflect clinically relevant network involvement rather than incidental remote changes.

The aim of this study was to assess whether retrospective SPM-assisted spatial normalization followed by AAL atlas-based ROI analysis of preoperative FDG-PET could help identify outcome-related metabolic differences among patients with similar routine presurgical classification, including concordant clinical, EEG, MRI, and visual FDG-PET findings. In particular, we asked whether extratemporal ROI-based metabolic measures, not emphasized in the original visual FDG-PET reports, differed according to surgical outcome beyond visually lateralized temporal hypometabolism in unilateral MRI-positive mTLE.

## 2. Materials and Methods

### 2.1. Patients

**Figure 1:**
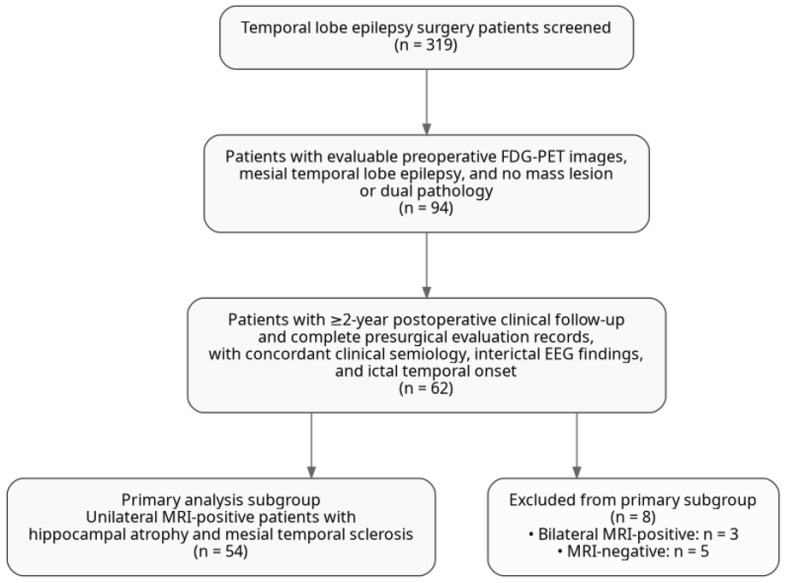
A total of 319 temporal lobe epilepsy surgery patients were screened. Among them, 94 had evaluable preoperative FDG-PET images, mesial temporal lobe epilepsy, and no mass lesion or dual pathology. Of these, 62 had at least 2 years of postoperative clinical follow-up and complete presurgical evaluation records, with concordant clinical semiology, interictal EEG findings, and ictal temporal onset. The primary quantitative analysis was restricted to 54 unilateral MRI-positive patients with hippocampal atrophy and mesial temporal sclerosis. The remaining 8 patients consisted of 3 bilateral MRI-positive and 5 MRI-negative cases.

This retrospective study screened 319 patients who underwent temporal lobe epilepsy surgery at Gazi University Faculty of Medicine over a 15-year period. Among them, 94 had evaluable preoperative FDG-PET images, and 62 patients aged 17 years or older had seizure-outcome data available at least 2 years after surgery and were included in the final study cohort. All included patients had drug-resistant mesial temporal lobe epilepsy (mTLE) and had undergone anterior temporal lobectomy. The diagnosis of mTLE was based on concordant presurgical data, including seizure semiology compatible with mesial temporal onset, long-term video-EEG findings supporting temporal seizure onset, epilepsy-protocol MRI findings, and visual FDG-PET interpretation. The primary quantitative FDG-PET ROI analysis was restricted to 54 unilateral MRI-positive patients with hippocampal atrophy and mesial temporal sclerosis on MRI. Patients with bilateral MRI-positive findings (*n* = 3) or MRI-negative scans (*n* = 5) were retained in the total cohort description but excluded from the primary ROI comparison analysis to reduce presurgical heterogeneity. The patient selection process is summarized in Figure 1.

All patients underwent standard anterior temporal lobectomy on the side indicated by the concordant presurgical evaluation. The procedure included resection of the anterior temporal neocortex together with mesial temporal structures, including the amygdala and hippocampus, according to the routine surgical protocol of the Department of Neurosurgery at Gazi University. No patient underwent a selective amygdalohippocampectomy or an extratemporal resection as part of the primary surgical procedure. In the postoperative period, antiseizure medication regimens were not changed during the first postoperative year unless clinically required. Afterward, one of the antiseizure drugs was gradually reduced in suitable patients. Antiseizure medication discontinuation did not affect outcome classification. Patients with recurrent postoperative seizures remained on antiseizure medication, and Engel classification was assigned according to seizure outcome at the 2-year clinical assessment, irrespective of medication status. The seizure freedom and postoperative antiseizure drug use of the patients were examined from the file records, and additionally, information was confirmed through recent phone calls. Files and records of patients who met these criteria were reviewed retrospectively, and demographic characteristics, clinical histories, background information, routine and VEM data, neuroimaging, psychiatric evaluations, and pathology results were collected. Existing FDG-PET/CT images of the patients were re-evaluated using SPM-assisted atlas-based ROI analysis. Postoperative seizure outcome was classified according to the Engel system using data obtained during the routine 2-year clinical follow-up visit by an experienced epileptologist. The epileptologist was blinded to the subsequent quantitative ROI reanalysis performed for this study. Patients with postoperative auras alone were classified as Engel I but not Engel IA. There were no missing data for the clinical, neuroimaging, pathological, and outcome variables included in the primary analyses and tables.

Our study was evaluated by the Gazi University Ethics Committee; ethics committee approval was received on 14 May 2019 with the decision number 2019-166.

### 2.2. Preoperative Protocol

Long-term video-EEG monitoring by a 32-channel digital EEG device was performed in all cases before surgery. For long-term monitoring, the electrodes were placed according to a 10–20 system, fixed on the scalp with collodion, and connected to a small amplifier on the patient. The amplifier was connected to the recording system (Information Recording Station) via a cable long enough to allow the patient to move freely in the room. With a video and sound system in the patient’s room, continuous image, sound, and simultaneous EEG recordings were provided by the computer system to which CD and DVD recorders were connected. Patients were monitored until at least three typical habitual seizures were recorded; the median number of recorded habitual seizures was 5 per patient. No additional electrodes were used, and none of the patients underwent invasive EEG monitoring. During the monitoring, the drugs were reduced and then discontinued in a controlled manner in order to observe the seizures. Lateralizing-localizing values of clinical findings were also determined by comparison with the surgery side.

All patients underwent a standard presurgical evaluation, including long-term video-EEG monitoring, epilepsy-protocol 3-Tesla MRI, functional MRI, FDG-PET/CT, and clinical assessment. Neuropsychological or psychiatric evaluations were reviewed when available, but they were not included in quantitative analyses because they were not standardized across the cohort. Histopathological reports were available for all patients. Hippocampal sclerosis subtypes were not specified in the original pathology reports. Cases classified as having nonspecific pathological findings showed gliotic or other nonspecific changes without sufficiently definite neuronal loss and sclerosis to establish a histopathological diagnosis of hippocampal sclerosis.

MRI classification was based on the official radiology reports and a dedicated review by a neuroradiologist who was a member of the study team. The neuroradiologist evaluated only the preoperative MRI findings and was blinded to postoperative seizure outcomes. Patients were classified as having unilateral MRI-positive disease when unilateral hippocampal atrophy and/or radiological features of mesial temporal sclerosis were present, with no structural abnormality identified in the contralateral mesial temporal region or elsewhere in the brain. Patients with bilateral mesial temporal abnormalities were classified as MRI-bilateral, whereas those without an identifiable structural epileptogenic abnormality were classified as MRI-negative.

### 2.3. FDG-PET Image Processing and ROI Analysis

At our center, the standard interictal brain FDG-PET protocol required a minimum 6 h fasting period before administration of 0.1 mCi/kg FDG. During the uptake phase, patients were instructed to remain still in a quiet, dimly lit room with their eyes open to minimize cerebral activation. Static brain PET images were acquired using a Discovery ST PET-CT camera (GE Medical Systems, GE Healthcare, Waukesha, WI, USA) 60 min after FDG injection. A CT scan was performed for attenuation correction, followed by a 15-min 3D PET emission acquisition. Images were reconstructed using an iterative algorithm.

Original visual FDG-PET reports were reviewed from clinical records. Visual interpretation was performed as part of routine presurgical evaluation by nuclear medicine physicians and was primarily directed toward temporal lateralization. For the present study, patients were classified according to whether the original report described unilateral temporal hypometabolism concordant with the surgical side. According to the institutional interictal FDG-PET practice, efforts were made to schedule scans outside the immediate peri-ictal period and to ensure at least 48 h of seizure freedom before tracer injection whenever clinically possible. Patients and/or caregivers were asked about recent seizures before tracer administration as part of routine clinical practice. Imaging was postponed if a peri-ictal state was clinically suspected or if a seizure occurred close to the scheduled scan. Continuous EEG monitoring was not performed during the uptake period, and sedative medications were not administered before or during imaging. However, because of the retrospective design, the exact seizure-free interval before FDG injection was not documented in a standardized manner for every patient and therefore could not be incorporated quantitatively into the analysis.

Quantitative analysis of preoperative brain [18F]FDG-PET images was performed using SPM8 (Wellcome Department of Cognitive Neurology, University College London, UK) and WFU PickAtlas 3.0.5 software (ANSIR Laboratory, Wake Forest University School of Medicine, USA) [8,9]. Patient PET images were exported from the camera system as axial PET slices in Interfile format and then converted into an analysis format readable by SPM. Spatial normalization of FDG-PET images to Montreal Neurological Institute (MNI) space was performed using a locally developed institutional FDG-PET brain template that conformed to the MNI atlas. Each normalized image was visually inspected to confirm adequate spatial normalization. After normalization, images were smoothed with a 12 mm full width at half maximum (FWHM) isotropic Gaussian kernel to improve the signal-to-noise ratio.

Regional metabolic values were extracted from the normalized FDG-PET images using the Automated Anatomical Labeling (AAL) atlas [10]. Eighty supratentorial and infratentorial brain regions were included in the ROI analysis. For each patient, mean count values were obtained for each ROI. To reduce interindividual variability in global tracer uptake, each regional value was normalized to the mean cerebellar uptake. Cerebellum-normalized uptake ratios were calculated as follows:

ROI uptake ratio = mean count of the ROI/mean cerebellar count.

These cerebellum-normalized ROI uptake ratios were used for all subsequent left/right and ipsilateral/contralateral statistical comparisons. In the present study, SPM was used for spatial normalization and preprocessing followed by atlas-based ROI extraction; no voxel-wise SPM group comparison or voxel-level corrected statistical mapping was performed.

The cerebellum was selected as the reference region because it is commonly used for count normalization in brain FDG-PET studies and lies outside the primary temporal-limbic and extratemporal cortical ROIs of interest. In addition, no focal cerebellar abnormality was identified on visual FDG-PET assessment or structural MRI review that would preclude its use as a reference region.

As a reference-region check, raw cerebellar uptake values were compared between Engel I and Engel II–IV outcome groups. In addition, to assess whether the ROI findings were dependent on cerebellar normalization, the main analyses were repeated using global mean normalization. For this sensitivity analysis, ROI values were recalculated using the global cerebral mean as the denominator, and the conventional left/right and ipsilateral/contralateral ROI comparisons were repeated using the same statistical approach.

### 2.4. Statistical Analysis

The primary outcome was defined as Engel class I versus Engel class II–IV at the 2-year postoperative follow-up. Analyses were first performed in the total cohort. Because MRI-negative and bilateral MRI-abnormality cases were clinically more heterogeneous, the primary quantitative FDG-PET ROI analyses were performed in the unilateral MRI-positive subgroup.

Continuous variables were presented as mean ± standard deviation and compared using the Mann–Whitney U test. Categorical variables were presented as counts and percentages. Fisher’s exact test was used for 2 × 2 tables, and the Fisher–Freeman–Halton exact test was used for larger contingency tables when expected cell counts were small. Linear-by-linear association was additionally evaluated for ordered categorical variables.

FDG-PET ROI values were compared between Engel I and Engel II–IV groups using the Mann–Whitney U test. Conventional left/right ROI analyses were performed first. Then, homologous ROIs were transformed into ipsilateral and contralateral values according to the side of anterior temporal lobe surgery. The conventional left/right ROI analysis and the ipsilateral/contralateral ROI analysis were treated as two distinct exploratory statistical families, each including 80 ROI comparisons. FDR correction was applied separately within each family. The ipsilateral/contralateral analysis was planned to account for the side of surgery. The left ATL subgroup analysis was considered an additional exploratory subgroup analysis and was interpreted descriptively rather than as a confirmatory analysis.

For ROI analyses, effect size was calculated as r = |*Z*|/√*N*. Hodges–Lehmann median differences with 95% confidence intervals were reported to summarize the magnitude and uncertainty of between-group differences. *p*-values from ROI comparisons were corrected using the Benjamini–Hochberg false discovery rate procedure. FDR-adjusted q < 0.05 was considered statistically significant. ROIs with unadjusted *p* < 0.05 but q ≥ 0.05 were interpreted as exploratory candidate regions rather than statistically robust biomarkers.

Because only nine patients in the primary unilateral MRI-positive subgroup had Engel II–IV outcomes, the study was not adequately powered for confirmatory ROI-based biomarker testing, robust multivariable logistic regression, or individual-level prediction modeling. Multivariable models were therefore not fitted to avoid severe overfitting and unstable parameter estimates. The ROI analyses were interpreted as exploratory group-level comparisons rather than as independent prognostic modeling. All tests were two-sided, and *p* < 0.05 was used only to identify unadjusted exploratory candidate differences before FDR correction.

## 3. Results

### 3.1. Demographic and Clinical Characteristics

Among 319 screened temporal lobe epilepsy surgery patients, 94 had evaluable preoperative FDG-PET images, a clinical phenotype compatible with mesial temporal lobe epilepsy, and no mass lesion or dual pathology. Of these, 62 patients had at least 2 years of postoperative clinical follow-up and complete presurgical evaluation records, with concordant clinical semiology, interictal EEG findings, and ictal temporal onset. The primary quantitative ROI analysis was restricted to 54 unilateral MRI-positive patients with hippocampal atrophy and mesial temporal sclerosis on MRI. The remaining 8 patients consisted of 3 bilateral MRI-positive and 5 MRI-negative cases and were retained only for descriptive characterization of the total cohort.

The association between MRI group and Engel outcome did not reach statistical significance (Fisher–Freeman–Halton exact test, *p* = 0.055). The higher proportion of Engel II–IV outcomes observed among MRI-negative patients may have been influenced by the small number of patients in the MRI-negative and bilateral MRI subgroups. Because MRI-negative and bilateral MRI-abnormality groups were small and showed a higher proportion of Engel II–IV outcomes, the primary quantitative FDG-PET ROI analyses were performed in the more homogeneous unilateral MRI-positive subgroup. Among patients with unilateral MRI-positive mTLE, 45 of 54 patients (83.3%) had an Engel I outcome, and 9 (16.7%) had an Engel II–IV outcome. More specifically, 30 patients were classified as Engel IA, 15 as Engel I non-IA, 7 as Engel II, and 2 as Engel III–IV.

### 3.2. Reference-Region Check and Global Mean Normalization Sensitivity Analysis

As a reference-region check, raw cerebellar uptake did not differ significantly between Engel I and Engel II–IV patients (*p* = 0.610), suggesting that the primary outcome comparison was not driven by a systematic imbalance in the cerebellar reference region. The global mean normalization sensitivity analysis did not materially change the overall interpretation. In the conventional left/right analysis, three ROIs showed unadjusted differences, left inferior orbital frontal cortex (1.130 [0.062] vs. 1.086 [0.033], *p* = 0.022, FDR q = 0.676), left medial orbital frontal cortex (1.203 [0.081] vs. 1.146 [0.042], *p* = 0.029, FDR q = 0.676), and right Heschl gyrus (1.238 [0.083] vs. 1.274 [0.088], *p* = 0.046, FDR q = 0.676), but none survived FDR correction.

### 3.3. Clinical Characteristics in the Unilateral MRI-Positive Subgroup

The unilateral MRI-positive group included 54 patients, of whom 45 had Engel I outcomes, and 9 had Engel II–IV outcomes. Age at surgery did not differ significantly between the groups (27.93 ± 7.50 vs. 30.00 ± 5.61 years, respectively; *p* = 0.254). Disease duration was also similar between Engel I and Engel II–IV patients (17.64 ± 7.76 vs. 17.56 ± 8.92 years; *p* = 0.816). The median number of recorded habitual seizures was 5 per patient.

Sex distribution (*p* = 0.461) and hemispheric language dominance (*p* = 0.403) were not significantly associated with outcome. Dominant-hemisphere surgery was performed in 21 of 45 Engel I patients (46.7%) and 7 of 9 Engel II–IV patients (77.8%), although the difference was not statistically significant (*p* = 0.297). Focal-to-bilateral tonic–clonic seizures were present in 33 Engel I patients (73.3%) and 8 Engel II–IV patients (88.9%; *p* = 0.428). Seizure frequency was also not significantly associated with outcome (*p* = 0.333).

Among the nine patients with Engel II–IV outcomes, the exact outcomes were Engel IIA in three patients, Engel IIB in four, Engel IIIB in one, and Engel IVA in one. Seven patients underwent left ATL and two underwent right ATL. All nine were left-language dominant; therefore, seven underwent dominant-hemisphere surgery and two underwent non-dominant-hemisphere surgery. MRI lateralization, predominant interictal EEG abnormalities, ictal EEG onset, and clinical semiology were concordant with the operated temporal lobe in all nine patients. Rapid contralateral propagation was not observed during the recorded seizures. The median postoperative follow-up duration in these patients was 46 months (range, 24–113 months).

Rare contralateral temporal interictal discharges were observed in six patients, all of whom were in the Engel I group. In these cases, contralateral sharp-wave or spike discharges accounted for less than 20% of the epileptiform discharges recorded during prolonged video-EEG monitoring. No Engel II–IV patient showed rare contralateral temporal interictal discharges.

Overall, five patients had nonspecific pathological findings, whereas the remaining patients had hippocampal sclerosis. Three nonspecific cases were in the unilateral MRI-positive subgroup, one was in the bilateral MRI-abnormality group, and one was in the MRI-negative group. Within the unilateral MRI-positive subgroup, only one of the three patients with nonspecific pathological findings had an Engel II–IV outcome (Table 1).

### 3.4. Conventional Left/Right ROI Analysis in the Unilateral MRI-Positive Subgroup

**Figure 2:**
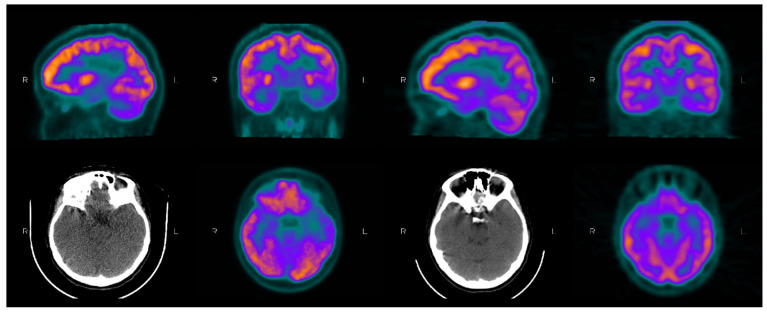
Representative preoperative FDG-PET/CT images from patients who underwent left and right anterior temporal lobectomy. The left panel shows left temporal hypometabolism in a patient who underwent left anterior temporal lobectomy, whereas the right panel shows right temporal hypometabolism in a patient who underwent right anterior temporal lobectomy.

The conventional left/right ROI analysis included 80 ROI comparisons in the unilateral MRI-positive subgroup. Ten ROIs showed unadjusted differences between Engel I and Engel II–IV patients. In all of these regions, Engel II–IV patients had lower FDG-PET uptake ratios than Engel I patients (Figure 2, Table 2).

The lowest unadjusted *p*-value was observed in the left inferior frontal triangular region. The median uptake ratio was 1.459 in Engel I patients and 1.365 in Engel II–IV patients, with a Hodges–Lehmann median difference of 0.088 (95% CI: 0.022 to 0.155; *p* = 0.014, r = 0.333). Other unadjusted differences were mainly observed in left frontal-orbitofrontal, medial frontal, middle cingulate, and inferior parietal regions. Hodges–Lehmann estimates were positive for all candidate ROIs, indicating lower cerebellum-normalized uptake ratios in Engel II–IV patients; however, confidence intervals were relatively wide and several lower bounds were close to zero. After correction for multiple ROI comparisons using the false discovery rate method, none of these left/right ROI findings remained statistically significant. The FDR-adjusted q value for these candidate ROIs was 0.342 (Table 2).

In the exploratory comparison of Engel IA and non-IA outcomes, unadjusted differences were observed in the right amygdala (*p* = 0.043) and left middle orbitofrontal cortex (*p* = 0.047). However, neither finding remained significant after Benjamini–Hochberg correction across 80 ROI comparisons (FDR q = 0.921 for both).

### 3.5. Ipsilateral/Contralateral ROI Analysis in the Unilateral MRI-Positive Subgroup

To account for the side of surgery, homologous left and right ROIs were transformed into ipsilateral and contralateral values according to the side of anterior temporal lobe surgery. This analysis also included 80 ROI comparisons. Eight ipsilateral or contralateral ROIs showed unadjusted differences between Engel I and Engel II–IV patients. In all of these regions, Engel II–IV patients again had lower FDG-PET uptake ratios than Engel I patients (Table 3).

The lowest unadjusted *p*-value was observed in the contralateral middle orbital frontal region. The median uptake ratio was 1.343 in Engel I patients and 1.255 in Engel II–IV patients, with a Hodges–Lehmann median difference of 0.109 (95% CI: 0.017 to 0.200; *p* = 0.021, r = 0.314). Other unadjusted differences were mainly observed in contralateral frontal-orbitofrontal regions, contralateral middle cingulate, contralateral inferior parietal cortex, and ipsilateral rectus/medial orbitofrontal regions. Hodges–Lehmann estimates were positive for all candidate ROIs, indicating lower cerebellum-normalized uptake ratios in Engel II–IV patients. However, confidence intervals were relatively wide, and none of these ipsilateral/contralateral ROI findings survived FDR correction. The FDR-adjusted q value for these candidate ROIs was 0.370.

### 3.6. Exploratory Left ATL Subgroup Analysis

An additional exploratory analysis was performed in the unilateral MRI-positive left ATL subgroup. This subgroup included 33 patients, of whom 26 had an Engel I outcome and 7 had an Engel II–IV outcome. Three orbitofrontal ROIs showed unadjusted differences between Engel I and Engel II–IV patients. The lowest unadjusted *p*-value was observed in the ipsilateral medial orbital frontal region, with a Hodges–Lehmann median difference of 0.090 (95% CI: 0.008 to 0.172; *p* = 0.027, r = 0.383). Additional unadjusted differences were observed in the ipsilateral superior orbital frontal region and contralateral superior orbital frontal region. Hodges–Lehmann estimates were positive for all three ROIs, indicating lower cerebellum-normalized uptake ratios in Engel II–IV patients. However, none of these left ATL subgroup findings remained significant after FDR correction. The FDR-adjusted q value was 0.544 (Table 4).

## 4. Discussion

The main purpose of this study was not to compare clearly different presurgical groups, but to examine whether quantitative FDG-PET could reveal additional metabolic differences among patients who were otherwise similarly classified before surgery. For this reason, the primary ROI analysis focused on unilateral MRI-positive mTLE patients with concordant clinical, EEG, MRI, and visually lateralized temporal PET findings. Patients with Engel II–IV outcome showed lower FDG-PET uptake ratios in several frontal, orbitofrontal, medial frontal, cingulate, and inferior parietal regions at the unadjusted level. However, none of these ROI findings survived FDR correction; therefore, these observations should be interpreted as exploratory candidate differences rather than statistically robust outcome-related PET markers.

The overall clinical structure of the cohort supports the decision to perform the primary ROI analysis in a more homogeneous unilateral MRI-positive subgroup. In the total cohort, 49 of 62 patients achieved Engel class I outcome, corresponding to a seizure-free rate of 79.0%, which is consistent with expected outcomes after surgery for mTLE when presurgical data are concordant. MRI-negative and bilateral MRI-abnormality cases showed a numerically higher proportion of Engel II–IV outcomes than unilateral MRI-positive cases; however, MRI-based grouping did not reach statistical significance in this cohort. Therefore, this observation was not interpreted as an independent predictor of outcome. Instead, it was used to support the analytic choice of restricting the primary quantitative FDG-PET analysis to unilateral MRI-positive patients, in whom clinical, EEG, MRI, and visual PET findings were more concordant. This approach is consistent with the broader literature indicating that multimodal concordance is associated with more favorable surgical outcomes in epilepsy surgery. In contrast, MRI-negative, discordant, bilateral, or diffuse/nonlocalizing metabolic patterns are associated with greater presurgical uncertainty [5,11,12].

Rare contralateral temporal interictal discharges were observed in six patients in the Engel I group and in none of the Engel II–IV patients. In these cases, contralateral discharges accounted for less than 20% of the recorded epileptiform discharges, whereas the predominant interictal abnormality, ictal EEG onset, clinical semiology, MRI lateralization, and visual PET lateralization were concordant with the operated temporal lobe. Therefore, rare contralateral interictal discharges were not enriched among patients with unfavorable outcome in this cohort. Nevertheless, because of the small number of Engel II–IV outcomes, this finding should be interpreted descriptively rather than as evidence that bilateral interictal activity has no prognostic relevance.

The MRI-positive patients with nonspecific pathological findings were considered separately from the EEG concordance issue. Three patients in the unilateral MRI-positive subgroup had nonspecific pathological findings rather than histopathologically definite hippocampal sclerosis, and only one of these had an Engel II–IV outcome. This discrepancy does not necessarily invalidate the preoperative MRI classification, because histopathological reports may be influenced by sampling, tissue orientation, processing-related limitations, or mild/atypical mesial temporal injury that does not meet full histopathological criteria for hippocampal sclerosis. However, it indicates that radiologically MRI-positive mTLE may still include residual histopathological heterogeneity. Therefore, these cases were retained in the unilateral MRI-positive analysis but are acknowledged as a potential source of outcome variability.

In the conventional left/right analysis, the unadjusted findings were predominantly left-sided and involved frontal-orbitofrontal, medial frontal, cingulate, and inferior parietal regions. This left-weighted pattern should not be interpreted as a new lateralizing PET sign, because the original visual reports had already classified the patients as having lateralized temporal hypometabolism. The anatomical distribution of the unadjusted candidate ROIs is therefore best considered a qualitative observation rather than evidence of statistically proven spatial clustering, as no formal spatial-clustering or permutation analysis was performed. In the subsequent ipsilateral/contralateral transformation, the biggest unadjusted differences shifted toward contralateral frontal-orbitofrontal regions, with additional ipsilateral medial/orbitofrontal and contralateral cingulate-parietal involvement. Nevertheless, the involvement of frontal-orbitofrontal, cingulate, and inferior parietal regions is biologically plausible in mTLE because these areas participate in temporal-limbic propagation pathways and broader network dysfunction [13,14,15,16,17]. They may help define network-level hypotheses for future adequately powered studies.

Our findings partly overlap with, but are not identical to, previous FDG-PET outcome studies in mTLE. Chassoux et al. reported that the best surgical outcome was associated with focal anteromesial temporal hypometabolism, whereas non-IA outcome was associated with more extensive extratemporal patterns. In their study, these patterns differed according to the side of hippocampal sclerosis, including mesial frontal and perisylvian hypometabolism in right HS and contralateral fronto-insular abnormalities in left HS [3]. Cahill et al. also emphasized lateralization-dependent metabolic patterns and found that contralateral temporal hypometabolism was associated with unfavorable outcome in right mTLE [4]. Kojan et al. found poor outcome-related hypometabolism mainly in the ipsilateral insula and contralateral temporal pole [18]. In comparison, our unadjusted candidate regions were more orbitofrontal, medial frontal, cingulate, and inferior parietal, rather than insular.

Several methodological and cohort-related differences may explain this discrepancy. First, our study was designed around a different clinical question. We did not primarily compare broad PET patterns between clearly different mTLE subgroups or between patients and healthy controls. Instead, we examined whether atlas-based ROI values differed between outcome groups among patients who had already been visually reported as having lateralized temporal hypometabolism. Conceptually, such a design may be suited to exploring subtle extratemporal group-level differences beyond the dominant temporal abnormality, but the small Engel II–IV subgroup in the present cohort precludes strong inference. Second, our main analysis was restricted to unilateral MRI-positive patients to reduce heterogeneity. This differs from studies that included broader HS cohorts, MRI-negative patients, or groups defined by complete seizure freedom versus non-IA outcome. Third, we used cerebellum-normalized AAL ROI values rather than voxel-level hypometabolic clusters or resection-volume-based measures. ROI averaging may reduce sensitivity to focal temporal or insular abnormalities but may highlight broader regional network effects. Fourth, the outcome group was small, and none of the findings survived FDR correction. Therefore, the orbitofrontal predominance observed in our study may be compatible with a network-level interpretation, but it should also be considered in light of the small outcome group and limited statistical power.

The repeated appearance of orbitofrontal regions is nevertheless biologically plausible. Orbitofrontal and medial frontal areas are closely linked to temporal-limbic structures through frontotemporal and limbic pathways. They may be involved in seizure propagation, chronic network dysfunction, or altered metabolic connectivity in mTLE. This interpretation is supported indirectly by metabolic network studies. Cho et al. showed that surgical outcome in TLE with hippocampal sclerosis was related to widespread FDG-PET network differences rather than a single temporal abnormality [6]. Strýček et al. also reported outcome-related metabolic connectivity changes involving temporal, insular, hippocampal, opercular, and orbitofrontal networks [7]. Our study did not assess metabolic connectivity directly, but the ROI pattern is compatible with the concept that unfavorable outcomes may be associated with a broader temporal-limbic-frontal network abnormality.

Another important point is that extratemporal hypometabolism should not be equated directly with temporal-plus epilepsy. In mTLE, extratemporal metabolic abnormalities may reflect seizure propagation, chronic network dysfunction, secondary synaptic depression, or compensatory network changes. Chassoux et al. emphasized that FDG-PET patterns in hippocampal sclerosis were closely related to electroclinical network organization rather than being explained only by structural MRI abnormalities [3]. In our cohort, the original visual interpretation remained temporal and lateralizing, whereas retrospective quantitative ROI analysis suggested additional extratemporal candidate differences. Because the main analysis was performed in a relatively homogeneous unilateral MRI-positive group, these findings are better interpreted as possible network-level metabolic differences within mTLE rather than as direct evidence of temporal-plus epilepsy. Importantly, these extratemporal ROI findings should not be interpreted as evidence of additional epileptogenic zones or as potential surgical targets. They do not support extending the temporal resection, modifying the surgical strategy, or exploring orbitofrontal, cingulate, or parietal regions in individual patients.

In the unilateral MRI-positive subgroup, age at surgery, disease duration, sex, hemispheric dominance, dominant-hemisphere surgery, focal-to-bilateral tonic–clonic seizures, and seizure frequency were also not significantly different between outcome groups. Dominant hemisphere surgery and focal-to-bilateral tonic–clonic seizures were numerically more frequent among Engel II–IV patients in some analyses, but these differences were not significant. Therefore, the exploratory FDG-PET findings were not clearly explained by these measured clinical variables. However, the small number of Engel II–IV outcomes prevented reliable multivariable modeling.

Several limitations should be acknowledged. First, this was a retrospective single-center study, and the number of patients with Engel II–IV outcomes was small, particularly in the unilateral MRI-positive and left ATL subgroup analyses. This limited statistical power and precluded robust multivariable modeling or reliable adjustment for potential clinical confounders. Accordingly, the unadjusted ROI findings should not be interpreted as independent predictors of surgical outcome or as corrected PET biomarkers.

Second, postoperative outcome classification has inherent limitations in a retrospective cohort. Although Engel class was assigned from structured clinical follow-up information, the Engel IA versus non-IA sensitivity analysis may have been influenced by the subjective nature of postoperative aura reporting. The non-IA group may include clinically different situations, ranging from subjective aura-like symptoms to disabling seizure recurrence, which increases outcome heterogeneity and may introduce reporting-related misclassification.

Third, because the cohort covered a 15-year retrospective period, some clinically relevant variables could not be standardized or fully incorporated into the analysis. Neuropsychological and neuropsychiatric assessments were not available in a sufficiently homogeneous format across the cohort. In addition, although the institutional interictal FDG-PET practice aimed to ensure at least 48 h of seizure freedom before tracer injection, the exact seizure-free interval was not documented in a standardized manner for every patient. Therefore, peri-PET seizure timing could not be included as a quantitative covariate, although recent seizure activity or postictal effects may influence FDG-PET uptake patterns, including extratemporal metabolic changes.

Fourth, the ROI-based approach may miss focal voxel-level abnormalities and does not directly assess metabolic connectivity. Cerebellar normalization is another methodological consideration. Although no focal cerebellar abnormality was identified on visual PET or structural MRI review and raw cerebellar uptake did not differ between outcome groups, reference-region effects cannot be fully excluded in FDG-PET studies. In particular, subtle remote cerebellar metabolic effects or network-related cerebellar involvement, even when not evident on routine structural MRI or visual PET assessment, could theoretically influence cerebellum-normalized cortical uptake ratios in temporal lobe epilepsy. The global mean normalization sensitivity analysis showed that the full unadjusted cerebellum-normalized ROI pattern was not preserved when an alternative denominator was used. Therefore, the present findings should be interpreted as cerebellum-normalized exploratory group-level differences rather than reference-independent prognostic PET biomarkers. Larger, externally validated cohorts with standardized peri-PET seizure documentation, harmonized neuropsychological assessment, reference-region sensitivity analyses, and network-based measures are needed before such findings can be applied to individual surgical decision-making.

Residual confounding also cannot be excluded. Potential confounders include epilepsy duration and severity, seizure burden, antecedent risk factors, antiseizure medication load, peri-PET seizure timing, surgical laterality, hemispheric dominance, MRI severity, electroclinical concordance, neuropsychological and psychiatric factors, and histopathological heterogeneity. Because only nine patients had Engel II–IV outcomes, robust multivariable adjustment was not feasible.

Despite these limitations, the study has several strengths. Rather than comparing clearly heterogeneous presurgical groups, the primary analysis focused on a relatively homogeneous unilateral MRI-positive mTLE subgroup with concordant clinical, EEG, MRI, and visually lateralized temporal FDG-PET features. The pathological profile of this subgroup was also relatively homogeneous, with hippocampal sclerosis in most patients and nonspecific pathological findings in only a small minority. This reduced, although it did not eliminate, the potential confounding effect of different underlying histopathologies on metabolic patterns and surgical outcome.

This design allowed us to pose a clinically relevant question: whether quantitative atlas-based ROI analysis could identify outcome-related metabolic differences among patients who appeared similar during routine presurgical evaluation. In the present dataset, the answer was limited: unadjusted group-level differences were observed, but the findings were not sufficiently robust to support reliable anticipation of an unfavorable outcome. Another strength is that the same preoperative FDG-PET scans were re-evaluated using both conventional left/right and ipsilateral/contralateral ROI approaches, together with reference-region sensitivity analyses. This structured reanalysis may help frame future studies examining whether extratemporal metabolic patterns have reproducible clinical relevance in larger and adequately powered mTLE cohorts.

## 5. Conclusions

In conclusion, this retrospective atlas-based ROI analysis identified uncorrected group-level FDG-PET differences between Engel I and Engel II–IV patients within a relatively homogeneous unilateral MRI-positive mTLE subgroup. Engel II–IV patients showed lower cerebellum-normalized uptake ratios in frontal-orbitofrontal, medial frontal, cingulate, and inferior parietal regions at the unadjusted level. However, none of the ROI findings survived FDR correction, and the small number of Engel II–IV outcomes precluded robust multivariable modeling or individual-level prediction. Interpretation is further limited by incomplete standardized documentation of peri-PET seizure timing and by the reference-region dependence of the unadjusted ROI findings. Therefore, the present findings do not provide sufficient evidence to recommend quantitative atlas-based ROI PET assessment as a clinical adjunct or warning tool in presurgical decision-making. These results should be interpreted as exploratory and hypothesis-generating. Larger, adequately powered cohorts with prospectively defined analytic strategies are needed before the potential clinical utility of quantitative FDG-PET analysis in presurgical evaluation can be determined.

## Figures and Tables

**Table 1:** Clinical characteristics according to 2-year Engel outcome in the unilateral MRI-positive subgroup.

Variable	Engel I (*n* = 45)	Engel II–IV Total (*n* = 9)	Engel II–IV Subclass Breakdown		*p*-Value
			Engel II (*n* = 7)	Engel III–IV (*n* = 2)	
**Clinical characteristics**					
**Sex, *n* (%)**					**0.461**
Female	28 (62.2)	4 (44.4)	4 (57.1)	0 (0.0)	
Male	17 (37.8)	5 (55.6)	3 (42.9)	2 (100.0)	
**Age at epilepsy onset, years**	10.66 ± 8.02	12.44 ± 8.72	13.43 ± 8.66	9.00 ± 11.31	**0.455**
**Age at surgery, years**	27.93 ± 7.50	30.00 ± 5.61	31.43 ± 5.32	25.00 ± 4.24	**0.254**
**Disease duration, years**	17.64 ± 7.76	17.56 ± 8.92	18.00 ± 9.83	16.00 ± 7.07	**0.816**
**Focal-to-bilateral tonic–clonic seizures, *n* (%)**					**0.428**
Absent	12 (26.7)	1 (11.1)	1 (14.3)	0 (0.0)	
Present	33 (73.3)	8 (88.9)	6 (85.7)	2 (100.0)	
**Seizure frequency, *n* (%)**					**0.333**
1–10 seizures/month	33 (73.3)	5 (55.6)	4 (57.1)	1 (50.0)	
11–20 seizures/month	10 (22.2)	3 (33.3)	2 (28.6)	1 (50.0)	
21–30 seizures/month	2 (4.4)	1 (11.1)	1 (14.3)	0 (0.0)	
**Recorded antecedent risk factor, *n* (%)**					**0.521**
None	8 (17.8)	2 (22.2)	2 (28.6)	0 (0.0)	
Febrile convulsion only	27 (60.0)	4 (44.4)	3 (42.9)	1 (50.0)	
Febrile convulsion plus other antecedent factor	7 (15.6)	3 (33.3)	2 (28.6)	1 (50.0)	
Other non-febrile antecedent factor only	3 (6.7)	0 (0.0)	0 (0.0)	0 (0.0)	
**Hemispheric dominance, *n* (%)**					**0.403**
Right	7 (15.6)	0 (0.0)	0 (0.0)	0 (0.0)	
Left	34 (75.6)	9 (100.0)	7 (100.0)	2 (100.0)	
Bilateral	4 (8.9)	0 (0.0)	0 (0.0)	0 (0.0)	
**Dominant hemisphere surgery, *n* (%)**					**0.297**
Dominant hemisphere operated	21 (46.7)	7 (77.8)	5 (71.4)	2 (100.0)	
Non-dominant hemisphere operated	20 (44.4)	2 (22.2)	2 (28.6)	0 (0.0)	
Bilateral dominance	4 (8.9)	0 (0.0)	0 (0.0)	0 (0.0)	
**Preoperative evaluation and laterality**					
**Surgical side, *n* (%)**					**0.456**
Right ATL	19 (42.2)	2 (22.2)	2 (28.6)	0 (0.0)	
Left ATL	26 (57.8)	7 (77.8)	5 (71.4)	2 (100.0)	
**MRI lesion side, *n* (%)**					**0.456**
Right hippocampal atrophy/MTS	19 (42.2)	2 (22.2)	2 (28.6)	0 (0.0)	
Left hippocampal atrophy/MTS	26 (57.8)	7 (77.8)	5 (71.4)	2 (100.0)	
**Visual PET hypometabolism side, *n* (%)**					**0.456**
Right temporal	19 (42.2)	2 (22.2)	2 (28.6)	0 (0.0)	
Left temporal	26 (57.8)	7 (77.8)	5 (71.4)	2 (100.0)	
**Ictal EEG onset side, *n* (%)**					**0.456**
Right temporal	19 (42.2)	2 (22.2)	2 (28.6)	0 (0.0)	
Left temporal	26 (57.8)	7 (77.8)	5 (71.4)	2 (100.0)	
**Ictal EEG onset matched operated side, *n* (%)**					-
Matched operated temporal lobe	45 (100.0)	9 (100.0)	7 (100.0)	2 (100.0)	
Not matched/unclear	0 (0.0)	0 (0.0)	0 (0.0)	0 (0.0)	
**Interictal EEG findings, *n* (%)**					**0.574**
Ipsilateral temporal only	39 (86.7)	9 (100.0)	7 (100.0)	2 (100.0)	
Rare contralateral temporal discharges (<20%)	6 (13.3)	0 (0.0)	0 (0.0)	0 (0.0)	
**PET-to-surgery interval, years**	0.58 ± 0.89	0.78 ± 0.67	0.71 ± 0.76	1.00 ± 0.00	**0.220**
**Postoperative follow-up duration, months**	54.69 ± 29.31	51.89 ± 29.02	44.71 ± 20.52	77.00 ± 50.91	**0.889**
**Pathological findings**					
**Pathological findings, *n* (%)**					**0.428**
Hippocampal sclerosis	43 (95.6)	8 (88.9)	6 (85.7)	2 (100.0)	
Nonspecific pathology	2 (4.4)	1 (11.1)	1 (14.3)	0 (0.0)	

**Note.** Values are presented as mean ± standard deviation or *n* (%). Percentages are column percentages. *p*-values compare Engel I and Engel II–IV total groups. *p*-values for 2 × 2 categorical variables were calculated using Fisher’s exact test. *p*-values for contingency tables larger than 2 × 2 were calculated using the Fisher-Freeman-Halton exact test because of small expected cell counts. Continuous variables were compared using the Mann–Whitney U test. *p*-values are descriptive and were not corrected for multiple comparisons. The Engel II and Engel III–IV columns are descriptive subclass breakdowns and were not separately tested. Other non-febrile antecedent factors included a history of complicated delivery, head trauma, or central nervous system infection. Abbreviations: MTS, mesial temporal sclerosis; PET, positron emission tomography; EEG, electroencephalography.

**Table 2:** Conventional left/right ROI analysis in the unilateral MRI-positive subgroup.

ROI	Engel I Median (IQR)	Engel II–IV Median (IQR)	Hodges-Lehmann Median Difference (95% CI)	*p*	FDR q	r
Frontal Inf Tri L	1.459 (0.116)	1.365 (0.136)	0.088 (0.022 to 0.155)	0.014	0.342	0.333
Frontal Sup Orb L	1.445 (0.135)	1.362 (0.182)	0.097 (0.025 to 0.164)	0.017	0.342	0.324
Frontal Med Orb L	1.521 (0.103)	1.416 (0.228)	0.105 (0.027 to 0.199)	0.017	0.342	0.324
Rectus L	1.493 (0.107)	1.414 (0.134)	0.081 (0.010 to 0.165)	0.022	0.342	0.311
Frontal Inf Orb L	1.433 (0.108)	1.342 (0.160)	0.101 (0.026 to 0.174)	0.025	0.342	0.305
Frontal Mid Orb L	1.331 (0.166)	1.297 (0.145)	0.110 (0.007 to 0.164)	0.032	0.342	0.292
Frontal Sup Med L	1.400 (0.124)	1.287 (0.244)	0.099 (0.008 to 0.200)	0.034	0.342	0.289
Cingulum Mid L	1.483 (0.136)	1.363 (0.188)	0.084 (0.007 to 0.159)	0.045	0.342	0.273
Frontal Inf Oper L	1.461 (0.124)	1.378 (0.148)	0.077 (0.001 to 0.146)	0.050	0.342	0.267
Parietal Inf L	1.481 (0.138)	1.366 (0.169)	0.072 (0.000 to 0.152)	0.050	0.342	0.267

Note. Engel I, *n* = 45, and Engel II–IV, *n* = 9; 80 bilateral ROI variables were tested. Values are median (IQR width). *p*-values are unadjusted Mann–Whitney U test *p*-values. FDR q values were calculated using the Benjamini–Hochberg correction across 80 ROI comparisons. Effect size r was calculated as |Z|/sqrt(*N*). Hodges-Lehmann median differences are presented as Engel I minus Engel II–IV with 95% confidence intervals. No ROI survived FDR correction.

**Table 3:** Ipsilateral/contralateral ROI analysis in the unilateral MRI-positive subgroup.

ROI	Engel I Median (IQR)	Engel II–IV Median (IQR)	Hodges-Lehmann Median Difference (95% CI)	*p*	FDR q	r
Frontal Mid Orb contra	1.343 (0.185)	1.255 (0.171)	0.109 (0.017 to 0.200)	0.021	0.370	0.314
Frontal Sup Orb contra	1.445 (0.162)	1.365 (0.168)	0.084 (0.014 to 0.156)	0.022	0.370	0.311
Frontal Inf Tri contra	1.408 (0.144)	1.329 (0.141)	0.087 (0.011 to 0.166)	0.022	0.370	0.311
Rectus ipsi	1.489 (0.112)	1.414 (0.134)	0.075 (0.007 to 0.149)	0.034	0.370	0.289
Cingulum Mid contra	1.476 (0.123)	1.402 (0.175)	0.074 (0.009 to 0.144)	0.034	0.370	0.289
Frontal Med Orb contra	1.524 (0.116)	1.477 (0.240)	0.088 (0.005 to 0.180)	0.036	0.370	0.286
Frontal Med Orb ipsi	1.506 (0.136)	1.416 (0.229)	0.082 (0.003 to 0.172)	0.040	0.370	0.280
Parietal Inf contra	1.495 (0.143)	1.406 (0.203)	0.090 (0.003 to 0.183)	0.042	0.370	0.276

Note. Homologous left/right ROIs were transformed according to the side of ATL into ipsilateral and contralateral values. Engel I *n* = 45 and Engel II–IV *n* = 9; 80 ROI variables were tested. Values are median (IQR width). *p*-values are unadjusted Mann–Whitney U test *p*-values. FDR q values were calculated using the Benjamini–Hochberg correction across 80 ROI comparisons. Effect size r was calculated as |Z|/sqrt(*N*). Hodges-Lehmann median differences are presented as Engel I minus Engel II–IV with 95% confidence intervals. No ROI survived FDR correction.

**Table 4:** Left ATL subgroup analysis after ipsilateral/contralateral transformation.

ROI	Engel I Median (IQR)	Engel II–IV Median (IQR)	Hodges-Lehmann Median Difference (95% CI)	*p*	FDR q	r
Frontal Med Orb ipsi	1.521 (0.105)	1.416 (0.180)	0.090 (0.008 to 0.172)	0.027	0.544	0.383
Frontal Sup Orb ipsi	1.433 (0.089)	1.362 (0.131)	0.086 (0.007 to 0.163)	0.035	0.544	0.368
Frontal Sup Orb contra	1.444 (0.128)	1.365 (0.115)	0.078 (0.008 to 0.153)	0.035	0.544	0.368

Note. This exploratory analysis was restricted to the unilateral MRI-positive left ATL subgroup. Engel I, *n* = 26, and Engel II–IV, *n* = 7; 80 ROI variables were tested. Values are median (IQR width). *p*-values are unadjusted Mann–Whitney U test *p*-values; exact significance was displayed for these tests in SPSS 22. FDR q values were calculated using the Benjamini–Hochberg correction across 80 ROI comparisons. Effect size r was calculated as |Z|/sqrt(*N*). Hodges-Lehmann median differences are presented as Engel I minus Engel II–IV with 95% confidence intervals. No ROI survived FDR correction.

## Data Availability

The data presented in this study are available from the corresponding author upon reasonable request. The datasets generated and/or analyzed during the current study are not publicly available due to ethical and privacy restrictions related to patient-level clinical and neuroimaging data.

## Abbreviations

The following abbreviations are used in this manuscript:

AAL	Automated Anatomical Labeling
ASM	Antiseizure Medication
ATL	Anterior Temporal Lobectomy
EEG	Electroencephalography
FDG-PET	Fluorodeoxyglucose Positron Emission Tomography
FDR	False Discovery Rate
MRI	Magnetic Resonance Imaging
mTLE	Mesial Temporal Lobe Epilepsy
ROI	Region of Interest
SPM	Statistical Parametric Mapping
VEM	Video-EEG Monitoring
